# Naturalistic neuroscience and virtual reality

**DOI:** 10.3389/fnsys.2022.896251

**Published:** 2022-11-17

**Authors:** Kay Thurley

**Affiliations:** ^1^Faculty of Biology, Ludwig-Maximilians-Universität München, Munich, Germany; ^2^Bernstein Center for Computational Neuroscience Munich, Munich, Germany

**Keywords:** virtual reality, naturalistic behavior, naturalistic neuroscience, ecological validity, animal behavior, behavioral neuroscience

## Abstract

Virtual reality (VR) is one of the techniques that became particularly popular in neuroscience over the past few decades. VR experiments feature a closed-loop between sensory stimulation and behavior. Participants interact with the stimuli and not just passively perceive them. Several senses can be stimulated at once, large-scale environments can be simulated as well as social interactions. All of this makes VR experiences more natural than those in traditional lab paradigms. Compared to the situation in field research, a VR simulation is highly controllable and reproducible, as required of a laboratory technique used in the search for neural correlates of perception and behavior. VR is therefore considered a middle ground between ecological validity and experimental control. In this review, I explore the potential of VR in eliciting naturalistic perception and behavior in humans and non-human animals. In this context, I give an overview of recent virtual reality approaches used in neuroscientific research.

## 1. Introduction

The arguably most important feature of natural behavior is active exploration and interrogation of the environment (Gottlieb and Oudeyer, 2018). External stimuli are not passively perceived. What is paid attention to is selected and specifically probed, reflecting the animals' motivations and needs. Moreover, natural environmental features and sensory cues are dynamic, multimodal, and complex (Sonkusare et al., 2019). This is in stark contrast to laboratory settings, which are characterized by numerous repetitions of the same imposed stimuli. These stimuli are often directed to only a single sense under simplified, artificial conditions and are disconnected from the animal's responses. Repetitions are important for behavioral modeling and the search for neural correlates and mechanisms, which both rely on trial-based averaging. It is nevertheless not surprising that results from laboratory experiments are of limited ecological validity and may not reveal the neural mechanisms underlying natural behavior (Krakauer et al., 2017; Dennis et al., 2021). Virtual reality (VR) may be part of a solution to this problem.

With VR, an artificial environment is simulated in which the user's actions determine the sensory stimulation, closing the loop between stimulation, perception, and action. A major motivation for the application of VR in neurophysiology is the desire to test behavior while recording with apparatuses that cannot be easily carried by the test subject or that require stability that cannot be achieved during free movement. The potential of VR, however, lies beyond this simple wish to fixate a behaving subject in place.

In connection with scientific VR-use, terms like *ecological* and *ethological validity* as well as *naturalistic conditions* are frequently voiced. In this regard, VR is considered to stand above traditional laboratory methods while maintaining a similar level of experimental control (e.g., Bohil et al., 2011; Parsons, 2015; Minderer et al., 2016; Krakauer et al., 2017; Lenormand and Piolino, 2022).

In the present article, I explore the potential of VR for evoking naturalistic perception and behavior and to promote the understanding of underlying brain function. I will give an overview of current VR technologies applied in neuroscience and use cases across different species, motivate why they are used and evaluate them in view of naturalistic neuroscience. To begin, let us briefly address the question: what is VR?

## 2. What is VR?

In his book, LaValle (2020) defines VR as: “Inducing targeted behavior in an organism by using artificial sensory stimulation, while the organism has little or no awareness of the interference.” This definition seems quite broad, but is flexible enough to embrace a variety of approaches, including those relevant to the present article. In a neuroscientific VR experiment, the participant experiences the stimulation of one or more senses to create the illusion of a “reality” that is intended by the researcher. Limited awareness seems less crucial for neuroscientific VR applications. However, one can argue that limited awareness is important for the feeling of presence in the artificial world, which as a result is treated as being natural—a basis for ecological validity.

What is missing from the above definition is that the virtual world is updated based on the user's behavior, providing an interactive experience (Bohil et al., 2011; Dombeck and Reiser, 2011; Naik et al., 2020). In terms of VR application in neuroscience, this narrowing of the definition is important because it distinguishes VR from simple sensory stimulation. The update is done in real time so that a *closed loop* is achieved between stimulation and behavior. For a real-time experience the update cycle needs to be sufficiently fast; how fast depends on the perceptual capabilities of the animal species and the sensory-motor system under investigation. Update delays can be increased parametrically depending on the research question. The most extreme case is the *open loop*, where the stimulation and the participant's actions are independent. Open loop corresponds to conventional stimulus conditions typically used in neuroscience studies.

## 3. Why using VR? And why for naturalistic neuroscience?

Typical motivations for using VR revolve around three different aspects: (1) multimodal stimulation with flexible and precise control, (2) interactivity instead of purely passive perception, and (3) the application of neural recording techniques that require particular mechanical stability. For naturalistic approaches, the first two points are the most important, but in a neuroscience context, the last is also relevant. I therefore discuss these three motivations next in view of their utility for naturalistic paradigms. Stimulus control and closed-loop methods have been steadily refined throughout the history of VR. For an account with regard to animal VRs and specifically rodent VRs used in research, the interested reader may be referred to Thurley and Ayaz (2017) and Naik et al. (2020). A general history of VR can be found in LaValle (2020). Finally, in this section I address the issue of *immersion*, i.e., the ability of a VR to draw the user in so that they feel present in it, which is closely related to achieving naturalistic conditions with VR.

### 3.1. VR provides flexible stimulus control

As a laboratory technique, VR benefits from the ability to perform experiments under precise control. This is what lets VR induce targeted behavior. Confounding and unintended influences although not completely excluded can be substantially reduced. VR is inherently flexible. It provides control over the complexity of the environment such as its size or the positioning of landmarks. Space restrictions, which can be a problem in the laboratory, do not exist in VR. Features can be easily and quickly altered without the participant noticing. One may, e.g., add or remove certain cues and test their contribution to a neural activity or a behavior. The manipulations can be done systematically and without influencing other components of the environment (Powell and Rosenthal, 2017). Also, stimuli may be provided that are unavailable in nature – although this speaks against the naturalistic use focused at in the present article. All of the above is hard to achieve in the field, where it is often less obvious which cues are attended to and which information is leveraged and which not; think, for instance, of investigating spatial navigation of primates in their natural habitat (De Lillo et al., 2014; Dolins et al., 2014, 2017; Allritz et al., 2022).

In the following, I will discuss paradigms of VR stimulus control utilized in specific areas of neuroscience research.

#### 3.1.1. Spatial cognition and navigation

The most obvious neuroscience use of VR is in the study of spatial perception and navigation (Bohil et al., 2011; Thurley and Ayaz, 2017). However, the advantage of VR for this purpose has been questioned since locomotion in traditional real world laboratory paradigms like foraging for food on linear tracks and in open field boxes is more natural than on a treadmill or alike (Minderer et al., 2016). In real arenas, information from the external world, e.g., from visual cues, and internally generated information, e.g., from moving body parts, are coordinated. In VR, these sources may not be aligned due to problems integrating simulation and tracking.

Such conflicts are likely the reason for the altered responses of space-encoding neurons in the rodent brain found in VR compared to real-world experiments. Head-fixed or body-fixed rodents do not receive normal vestibular input, resulting in mismatches between vestibular and visual information. Place cells in the hippocampus show altered position coding under such conditions, as confirmed by direct comparisons between virtual paradigms and their real-world counterparts (Chen et al., 2013; Ravassard et al., 2013; Aghajan et al., 2014). In VR setups that do not restrict body rotations and in which vestibular information about rotational movements is available to the animal, normal place-selective firing has been reported (Aronov and Tank, 2014; Chen et al., 2018; Haas et al., 2019). Freely-moving VRs may even better solve this problem (Del Grosso et al., 2017; Kaupert et al., 2017; Stowers et al., 2017; Madhav et al., 2022).

Thus, the design of a VR setup and the quality of a VR simulation may elicit atypical neural responses. These issues do not devalue certain VR systems—each setup may provide informative insights—but they are important indications that the suitability for understanding natural behavior and associated neural activity may be limited for some VR applications.

However, VR has advantages over traditional laboratory paradigms, which are themselves far from the situation animals face in the wild. These advantages depend on the research question. The possibility to simulate environments that are much larger than the available space in the laboratory, can increase ecological validity (Dolins et al., 2017), e.g., for the study of spatial learning in macaques (Taillade et al., 2019) and chimpanzees (Allritz et al., 2022), and fly search behavior (Kaushik et al., 2020). Specialized VR systems and paradigms can provide insights into specific topics, (e.g., path integration Petzschner and Glasauer, 2011; Lakshminarasimhan et al., 2018, 2020; Thurley and Schild, 2018; Jayakumar et al., 2019; Robinson and Wiener, 2021; Madhav et al., 2022). VR enables task standardization for cross-species comparison. For instance, spatial behavior can be tested with humans in typical rodent laboratory mazes like the Morris water maze (Laczó et al., 2010). Moreover, VR helps to overcome difficulties of testing spatial behavior and cognition in the wild as I already pointed out above.

#### 3.1.2. (Multi-)sensory processing

In VR, several senses can be stimulated at once and in concert. Such a multimodal stimulation increases immersion and engagement. The experience will be more ecological and if natural stimuli are used more naturalistic. Already the first applications of VR, e.g., for studying sensory-motor control of flying in insects, combined visual, mechanosensory (wind source), and olfactory cues (Gray et al., 2002). In general, any VR method that connects locomotion with some type of sensory stimulation provides a multimodal experience because it inevitably encompasses sensory feedback about self-motion. In this sense, the most typical VR that uses visual stimulation with walking on a treadmill or tethered flying will always be multimodal.

In principle, unnatural stimuli may be given or the stimulation of different senses may be mismatched, allowing for experiments that are not possible in the real world. Of course, this speaks against the naturalistic principle, but let me nevertheless give a few examples for illustration. VR makes it possible to decouple stimuli that are inextricably linked in the real world. In rodent experiments, visual sensory have been dissociated from non-visual self-motion inputs to probe their differential influences on spatial responses in the hippocampal formation (Chen et al., 2013; Tennant et al., 2018; Haas et al., 2019; Jayakumar et al., 2019) or on running speed responses in visual cortex (Saleem et al., 2013). In flies, the feedback from eyes and halteres has been decoupled in simulated flight setups (Sherman and Dickinson, 2003). Also the lag between an action and the subsequent update of the virtual stimulation could be changed. For instance, positional changes could be delayed or made jump-/teleportation-like (see Domnisoru et al., 2013; Kaupert et al., 2017; Stowers et al., 2017; Tennant et al., 2018, for examples from experiments in fish and rodents). Thus, in general, sensory and motor variables can be separated in VR.

#### 3.1.3. Social interactions

The issues of laboratory vs. field work also apply to the study of social interactions. VR can alleviate some of them. Virtual stimuli can be designed to appear more similar to real-life counterparts than stimuli used in classical ethological experiments (Naik et al., 2020). A particular advantage is that stimuli can be animated. Even under open-loop conditions, moving prey can be simulated to study prey-capture (Ioannou et al., 2012) or conspecifics to probe mate-choice (Gierszewski et al., 2017). Further examples can be found in Naik et al. (2020).

An important point for experiments on social interactions is consistency (Powell and Rosenthal, 2017). No matter if in the wild or the laboratory, the behavior of real subjects depends on their motivation and will change by their interaction with others. Simulated subjects do not change their behavior in this manner (Chouinard-Thuly et al., 2017). Alike other VR stimuli, socially-relevant ones can also be precisely controlled, held constant or adapted, presented several times and to different subjects. Importantly, not just the static appearance is under close control but also the simulated movement patterns. In general, interaction with moving objects may sometimes be easier simulated in VR than provided in the real world, cf. e.g., the prey-capture study mentioned above (Ioannou et al., 2012).

### 3.2. VR goes beyond mere stimulus delivery

With VR the loop between perception and action can be closed. The participant in a VR experiment not only passively perceives the stimulation but behaves and interacts with it, which in turn changes the stimulus environment. This active engagement makes the VR experience much more reminiscent of real life and natural conditions than traditional, passive approaches.

The importance of motor actions for perception has been demonstrated, for instance, by experiments with mice moving on a treadmill while perceiving visual stimuli. Even when the treadmill is not coupled to the stimulation in such experiments, i.e., open-loop, neuronal responses in the visual cortex are substantially modulated (Niell and Stryker, 2010; Ayaz et al., 2013). More recently impacts of movement have been described not only for vision (Dadarlat and Stryker, 2017; Clancy et al., 2019) but also for audition and somatosensation (Fu et al., 2014; Schneider and Mooney, 2018). Similar dependence of sensory processing on behavioral state has also been reported in insects (Maimon et al., 2010). In zebrafish, the interaction between motor responses and visual feedback (Portugues and Engert, 2011) and related neural processing (Ahrens et al., 2012) has been investigated with closed-loop experiments as well as visually-driven swim patterns underlying natural prey capture (Trivedi and Bollmann, 2013).

Closed-loop is also helpful for decision making studies in species such as mice (Harvey et al., 2012), gerbils (Kautzky and Thurley, 2016), and zebrafish (Bahl and Engert, 2020; Dragomir et al., 2020). These studies use rather abstract visual stimuli, like random dots and stripe patterns, which are admittedly not very naturalistic. However, enabling natural motor responses, like walking and swimming, are key improvements over conventional designs that rely on nose-poking or lever-pressing.

### 3.3. VR enables recording of brain activity with bulky devices

One of the early motivations of using VR in neuroscience was that neural recording techniques can be used that require a high degree of mechanical stability or are too bulky and heavy to be carried by the animal (Dombeck et al., 2010; Harvey et al., 2012; Ahrens et al., 2013; Domnisoru et al., 2013; Schmidt-Hieber and Häusser, 2013; Leinweber et al., 2014). Similar reasons apply to the use of VR with fMRI or other methods for recoding human brain activity (Lenormand and Piolino, 2022). The application of VR with a focus on recording brain activity in the behaving animal has, e.g., been reviewed by Dombeck and Reiser (2011). Technological progress will increasingly weaken this motivation to use VR in the future. Miniature head-mounted systems for imaging (Yu et al., 2015) and single cell recordings (Valero and English, 2019) are under constant development and can be applied in freely moving animals.

### 3.4. Achieving immersion and presence in VR

Important concepts that are frequently expressed, especially in the context of human VR, are those of *immersion* and *presence*. How immersive a VR is, i.e., how strongly it draws the user in, is determined by the degree of sensory stimulation and the sensitivity to motor actions of the VR system in use. Deeper immersion leads to increased presence, i.e., the feeling of being in the virtual world (Bohil et al., 2011). For recreational or therapeutic applications with humans, high levels of immersion are surely desired and necessary. But how about scientific use?

Immersion does not seem to be the most important factor for investigating certain research questions. A VR setup could in principle only be a tool to provide some sensory stimulation and to connect it to behaviors. However, the ultimate goal of neuroscience is to investigate behavioral and brain responses that occur under natural conditions (Krakauer et al., 2017). A VR approach could only contribute to this goal if it elicits such responses. Yet, a VR that evokes responses as in real life implies deeper immersion. Thus, ecological validity and immersion are linked.

But how to determine presence and immersion? Humans can be questioned (Hofer et al., 2020), but what about animals? To determine and quantify the degree of immersion, two different types of responses seem at hand: neural and behavioral. However, not all possible types of neural activity must occur in natural behaviors, so only behavioral responses are suitable to determine proximity to natural conditions (Krakauer et al., 2017). Therefore, sufficient understanding of the behavior to be elicited in VR is required under real-world conditions.

A number of studies compared physiological and psychological reactions between real-life situations and their virtual counterparts in humans (examples are reviewed by Lenormand and Piolino, 2022). Behavioral conformity between virtual and real world is less regularly assessed with animals (Powell and Rosenthal, 2017). While it is more common in insects (see Dahmen et al., 2017 for an elegant example with ant spatial navigation) and spiders (Peckmezian and Taylor, 2015), work with rodents on this topic is scarce (Hölscher et al., 2005). Comparisons have instead been made of neural activity between real-world and virtual conditions (e.g., for space-responses in rodent hippocampus Chen et al., 2013; Ravassard et al., 2013; Safaryan and Mehta, 2021).

Immersion is often considered in the context of the quality of the visual stimulation as it is the dominant sensory modality in primates and flying insects. For other species, however, vision is not as dominant. In these animals, immersion and ecological validity will depend more strongly on other types of perceptions, e.g., sound, touch, smell. As an example that may not seem like VR at first glance but is nonetheless consistent with the broad definition of VR favored in this article and which is ecologically valid see Faumont et al. (2011). In this study, osmo-sensitive neurons of the nematode *Caenorhabditis elegans* were optogenetically activated to simulate an aversive location in the animal's environment.

## 4. Technical components for naturalistic VR

Key technical components of VR are devices that provide sensory stimulation to create the virtual experience and those that keep track of the behavioral responses. How these components may promote naturalistic stimulation and behavior is discussed next.

### 4.1. Tracking movements and actions

In VR setups, participants are often restrained so that they can sense the stimuli appropriately while having enough freedom to move in the virtual environment. For instance, a specific position may need to be maintained in relation to a screen for visual stimulation or speakers for auditory stimulation. Other reasons are requirements on mechanical stability of neural recording devices as was already discussed above.

The type of fixation depends on the tested species. Flying insects may be tethered with their body leaving the wings free to beat (Gray et al., 2002; Sherman and Dickinson, 2003; Dombeck and Reiser, 2011). Wing motion is monitored with an optical sensor, and the difference between the amplitudes of left and right wing beats serves as an indicator of attempted body rotations (Reiser and Dickinson, 2008). In legged animals, fixation on a treadmill is the standard technique (Carrel, 1972; Dahmen, 1980; Seelig et al., 2010; Takalo et al., 2012; Peckmezian and Taylor, 2015; Thurley and Ayaz, 2017; Haberkern et al., 2019; Naik et al., 2020). Such treadmills are typically styrofoam balls on an air-cushion, cylindrical treadmills or linear belts. The animals move the treadmill with their legs, which is captured and used to update the position in the virtual world. In animals like rodents, which have a natural need for walking (Meijer and Robbers, 2014), a treadmill gives a more natural way of responding to the animals—even in non-spatial tasks (Garbers et al., 2015; Kautzky and Thurley, 2016; Henke et al., 2021, 2022). To provide a realistic, natural feeling of motion, the physical properties of the treadmill, such as its moment of inertia, must be taken into account and adapted to the animal species. For instance, treadmills for ants have particularly low friction and weight (Dahmen et al., 2017).

Any type of fixation imposes unnatural movements and disrupts sensory feedback about motor behavior. Tethered insects do not receive normal input from their balance organs (Fry et al., 2008). Head-fixed rodents do not receive natural input about rotations and linear acceleration from their vestibular organs. Also they have to make unnatural shear movements with their legs on the treadmill to make rotations in the virtual environment (Thurley and Ayaz, 2017). A similar lack of vestibular input is also found in zebrafish VRs, in which the animals' heads are immobilized (e.g., Portugues and Engert, 2011). A solution to this problem is offered by VR setups for freely flying, walking, and swimming animals (Fry et al., 2008; Del Grosso et al., 2017; Stowers et al., 2017; Ferreiro et al., 2020; Madhav et al., 2022). These setups use cameras to track the position of the animal (or only its head) and update a perspective-correct visual scenery. Alternatively, tracking information can be used to drive a motorized treadmill that compensates for the animal's movements to hold it in place with respect to the VR hardware (Kaupert et al., 2017).

Several technical considerations apply to ensure proper tracking, especially to meet the needs of the experimental animal (see Naik et al., 2020). A number of different tracking methods exist based on deep learning and other machine learning techniques (e.g., Hedrick, 2008; Robie et al., 2017; Graving et al., 2019; Mathis and Mathis, 2020; Vagvolgyi et al., 2022).

Body fixation is also not required when the stimulus display is directly attached to the sense organ and can be carried as with head-mounted displays. In VR headsets, head-mounted displays are combined with head-tracking hardware (Bohil et al., 2011; LaValle, 2020). Headsets prevail in human VR nowadays but also other tracking methods exist like treadmills for humans (examples are found in LaValle, 2020). In humans and other primates, often joysticks, game pads or keyboards are used to track motion and other responses (Washburn and Astur, 2003; Sato et al., 2004), for instance, when particular fixation is necessary like in fMRI (Lenormand and Piolino, 2022).

### 4.2. Displaying visual stimuli

Visual virtual worlds are the predominant type of VR. They are almost exclusively provided in first-person view, i.e., from the point of view of the participant. Compared to a third person perspective behind a visible avatar—which might be possible with humans but is hard to imagine with animals—the first-person view enhances the experience (Dolins et al., 2017). For presentation, different types of displays are used, such as simple monitors, panoramic projection screens and head-mounted displays. Projections to the floor below the animals are also leveraged, e.g., with zebrafish (Ahrens et al., 2012; Bahl and Engert, 2020; Dragomir et al., 2020). In insects with their lower visual acuity but fast reaction times, LED displays are used (Dombeck and Reiser, 2011). For animals with eyes in the front, like primates and carnivorans, flat monitors may be sufficient. For animals with laterally positioned eyes, like rodents, only wide displays cover a sufficient part of the field of view (Dolins et al., 2017; Thurley and Ayaz, 2017). For ecological validity, the projection needs to have correct perspective and be undistorted (Dolins et al., 2017; Naik et al., 2020).

In general, it has to be kept in mind that images shown on displays are perceived differently by different animals (Chouinard-Thuly et al., 2017; Naik et al., 2020). Photoreceptor sensitivities differ across species (e.g., Osorio and Vorobyev, 2005) and the color display has to be adapted to the species' specifics to enable naturalistic stimulation. Behavioral methods can also readout animals' sensitivities (Knorr et al., 2018). Other visual capabilities like integration times and acuity also vary between species and need to be accommodated. For a detailed discussion with a focus on technical challenges see Naik et al. (2020). Similar considerations obviously apply to other sensory systems as well and have to be taken into account, especially when a naturalistic perceptual experience is intended.

Images on a screen remain 2D and natural vision is only partially achieved (Dolins et al., 2017). For instance, stereopsis is not possible with single images. Head-mounted displays in humans solve this by presenting offset images to each eye (LaValle, 2020). For an approach with insects, see Nityananda et al. (2016). Currently, there are no VR headsets for animals, although they may be in development, as they are mainly a miniaturization issue, apart from species-specific needs. Technology in this direction includes head-mounted camera systems to track eye movements (Meyer et al., 2018) and inertial sensors for head-tracking in rodents (Venkatraman et al., 2010; Fayat et al., 2021).

### 4.3. Sound stimulation

To simulate 3D spatial sound scenes that mimic real-life situations, virtual acoustic approaches have been developed. Human VR headsets often include headphones to provide sound stimuli in conjunction with the visual display. Alternatively, in free-field auralization, arrays of loudspeakers are placed around the user, such that sound sources can be precisely positioned in virtual space (Seeber et al., 2010). Compared to headphones, the user can listen with their own ears and the characteristics of their ears can be captured. Therefore, experiments can be also done with hearing aid wearers. A disadvantage is that the setup has to be placed in an anechoic chamber, which is demanding and expensive to construct. For correct deliverance of sound cues, the user has to be placed in a specific location with respect to the array. With such auditory VR setups, e.g., auditory motion parallax could be demonstrated in humans (Genzel et al., 2018). In rodents, virtual acoustics is done with loudspeakers placed around the treadmill (Cushman et al., 2013; Funamizu et al., 2016). Other approaches use more of an augmentation of a real arena than virtual acoustics to probe spatial localization of objects with the help of acoustic stimulation (Ferreiro et al., 2020; Amaro et al., 2021).

### 4.4. Tactile and haptic stimulation

In VR tactile and haptic stimulation can also be provided, simulating surfaces with different textures or the feel of forces (Bohil et al., 2011). Haptic systems for humans consist, e.g., of robotic arms with which force or pressure can be applied or pin arrays can be used to simulate surfaces (Culbertson et al., 2018; Wang et al., 2019). In tactile VR systems for rodents, the animals move through corridors simulated by movable plates (Sofroniew et al., 2014) or rotating cylinders with different textures (Ayaz et al., 2019). These “walls” are touched by the animals with their whiskers and they are adapted in closed-loop by the movements of the animal. Similar setups exist in which the animals are freely moving and that are not actually VR but still allow for simulating different tactile textures (Kerekes et al., 2017). Belt treadmills can also be equipped with tactile cues (Geiller et al., 2017).

### 4.5. Odors

Recently, devices have been developed to quickly and precisely deliver odorants with sufficient diffusion and clearance times for simulating spatially confined olfactory cues. Examples for use with humans are Salminen et al. (2018) and Micaroni et al. (2019). In animal studies, olfactory VR has been used with tethered rodents (Radvansky and Dombeck, 2018; Fischler-Ruiz et al., 2021; Radvansky et al., 2021) and insects (Gray et al., 2002). Precise odor delivery poses a problem for freely moving VRs, either a distribution system has to be carried on the body or, alternatively, odors could be delivered on room scale (Fry et al., 2008). However, the latter is hard to control in terms of odor concentration and distribution, preventing proper localization. Systems for humans that simulate taste are under development (Narumi et al., 2011; Vi et al., 2017; Kerruish, 2019) but have not yet been used in neuroscience as far as I know.

### 4.6. Rotation and gravity

VR setups that require fixation of the animals typically suffer from providing only inadequate information about rotational and linear acceleration cues. To overcome such problems, motion platforms with multiple degrees of freedom or rotating chairs providing horizontal rotations have been used for vestibular stimulation (Gu et al., 2010; Dokka et al., 2011; Genzel et al., 2016; Garzorz and MacNeilage, 2017). Similarly, rotational gimbals have been used with flies (Sherman and Dickinson, 2003). VR setups that allow for free movement do not suffer from these problems.

## 5. Limitations and potentials for naturalistic VR

Technical considerations for VRs with respect to species specifics and naturalistic experiments have already been discussed above. Here I address some more general issues.

### 5.1. Not everything can be tested in VR in terms of naturalistic experiments

There is in principle no limitation on what can be simulated with VR. For the purpose of the present article the simulation just needs to be naturalistic. We can intuitively judge how a VR simulation affects a human participant—or often simply take it for granted that we can—but this is impossible with animals. Thus, as I have argued above, naturalistic approaches must ensure that a VR simulation elicits the same behaviors that would occur in the real world counterpart (Krakauer et al., 2017; Powell and Rosenthal, 2017). This strongly constrains what can and cannot be done with VR in terms of naturalistic experiments. When a strict comparison between the real world and VR is not possible, such as with the teleportation-like position changes mentioned above, it means that the experiment is not suitable for a naturalistic VR study. Other questions may be better investigated directly in the real world, instead of investing in building a VR with all its limitations.

### 5.2. How natural can VR become and how natural or real does it have to be?

As pointed out by LaValle (2020), it is tempting to try to match the physical world in VR as closely as possible (*universal simulation principle*). Such a goal is inappropriate, since a simulation will never be perfect and always comprise unanticipated confounding variables. One should rather be guided by the research objective when designing the VR. A sensible design can at times mean reduction and simplification, without losing ecological validity (Bucci-Mansilla et al., 2021). Related to this is the *uncanny valley* phenomenon, in which high realism of an artificial stimulus makes observers feel uneasy (Chouinard-Thuly et al., 2017; LaValle, 2020). Among non-human animals this problem has been described with macaques (Steckenfinger and Ghazanfar, 2009).

### 5.3. VR sickness and fatigue

A regularly encountered problem with human VR applications is that of cyber, simulator, or *VR sickness* (Bohil et al., 2011; LaValle, 2020). Some participants experience discomfort and nausea due to latencies in the synchronization of the VR components, which results in incongruent sensory inputs. Of particular importance here is vestibular feedback from self-motion, which does not match visual input. This problem may occur due to improper tracking but also a misunderstanding and disregard of the user-perspective by the designer of the VR experience (LaValle, 2020). Related to this, *fatigue* can arise. Whereas, fatigue is certainly an issue that can be accounted for in animal studies—consider, for example, a treadmill that is too heavy or creates much friction (Dahmen et al., 2017)—analogs of VR sickness in animals may be difficult to determine. Animal VRs can suffer from unnatural feedback from different senses (Dombeck and Reiser, 2011; Thurley and Ayaz, 2017). This is exemplified by the issues of head-fixation with regard to hippocampal space-related activity in rodents discussed above.

## 6. Conclusions

In this article, I tried to show that VR has a multitude of applications in neuroscience that can help advancing from traditional laboratory-based to naturalistic research themes. VR can mediate between the opposing poles of ecological validity and experimental control, facilitating generalizability of laboratory results to the situation in the wild. As with any scientific approach, the means have to be adapted to the research question. A specific and maybe novel technology or method does not help with this by itself (Minderer et al., 2016; Thurley and Ayaz, 2017). When designing a VR, it is important to consider the specifics of the model species. Only then immersion can be reached, which results in a naturalistic experience and ecological validity. To determine how immersive a VR experience is, only behavioral readout is appropriate, which needs to be compared to real-world behavior. Otherwise, VR experiments will likely elicit unnatural behaviors and neural responses, which are not related to the intended research questions (Krakauer et al., 2017; Powell and Rosenthal, 2017).

Developers of VR for humans, especially for consumer applications or therapy, realized that without knowledge about our senses, our perception and ultimately our brains, it is not possible to build VR (LaValle, 2020). This concept closes the cycle for the present article—and presents a somewhat circular argument for use of VR in naturalistic neuroscience: VR is used in neuroscience to gain insights into perception, behavior, and brain function. However, good VR experiments that are also naturalistic and ecologically valid can only be conducted if the subjects' perception, behavior, and knowledge of their physiological basis are sensibly taken into account.

## Author contributions

The author confirms being the sole contributor of this work and has approved it for publication.

## Conflict of interest

The author declares that the research was conducted in the absence of any commercial or financial relationships that could be construed as a potential conflict of interest.

## Publisher's note

All claims expressed in this article are solely those of the authors and do not necessarily represent those of their affiliated organizations, or those of the publisher, the editors and the reviewers. Any product that may be evaluated in this article, or claim that may be made by its manufacturer, is not guaranteed or endorsed by the publisher.
